# Identification and characterization of amphibian SLC26A5 using RNA-Seq

**DOI:** 10.1186/s12864-021-07798-6

**Published:** 2021-07-22

**Authors:** Zhongying Wang, Qixuan Wang, Hao Wu, Zhiwu Huang

**Affiliations:** 1 Department of Otolaryngology-Head and Neck Surgery, Shanghai Ninth People’s Hospital, Shanghai Jiao Tong University School of Medicine, Shanghai, China; 2 Ear Institute, Shanghai Jiao Tong University School of Medicine, Shanghai, China; 3 Shanghai Key Laboratory of Translational Medicine on Ear and Nose Diseases, Shanghai, China

**Keywords:** RNA-Seq, SLC26A5, Prestin, Amphibian hearing organ, Non-linear capacitance (NLC), 3D protein structure

## Abstract

**Background:**

Prestin (SLC26A5) is responsible for acute sensitivity and frequency selectivity in the vertebrate auditory system. Limited knowledge of prestin is from experiments using site-directed mutagenesis or domain-swapping techniques after the amino acid residues were identified by comparing the sequence of prestin to those of its paralogs and orthologs. Frog prestin is the only representative in amphibian lineage and the studies of it were quite rare with only one species identified.

**Results:**

Here we report a new coding sequence of SLC26A5 for a frog species, *Rana catesbeiana* (the American bullfrog). In our study, the SLC26A5 gene of *Rana* has been mapped, sequenced and cloned successively using RNA-Seq. We measured the nonlinear capacitance (NLC) of prestin both in the hair cells of *Rana*’s inner ear and HEK293T cells transfected with this new coding gene. HEK293T cells expressing *Rana* prestin showed electrophysiological features similar to that of hair cells from its inner ear. Comparative studies of zebrafish, chick, *Rana* and an ancient frog species showed that chick and zebrafish prestin lacked NLC. Ancient frog’s prestin was functionally different from *Rana*.

**Conclusions:**

We mapped and sequenced the SLC26A5 of the *Rana catesbeiana* from its inner ear cDNA using RNA-Seq. The *Rana* SLC26A5 cDNA was 2292 bp long, encoding a polypeptide of 763 amino acid residues, with 40% identity to mammals. This new coding gene could encode a functionally active protein conferring NLC to both frog HCs and the mammalian cell line. While comparing to its orthologs, the amphibian prestin has been evolutionarily changing its function and becomes more advanced than avian and teleost prestin.

## Background

Prestin is localized in the lateral wall of outer hair cells (OHCs) and is a membrane-based motor protein that powers electromotility. Electromotility as a central mechanism in the mammalian inner ear is unique to the OHCs and absent in the inner hair cells (IHCs) [1]. This mechanical activity is believed to feed back into the vibration of the cochlear partition, thereby enhancing the mechanical stimulus of IHCs [2]. SLC26A5 inactivation in mammals resulted in a loss of OHC somatic motility in vitro and a 40–60 dB loss of cochlear sensitivity in vivo [1]. OHC electromotility has several salient features, and obtains its energy supply via changing membrane potential instead of ATP hydrolysis. Also, although internal Ca^2+^ levels modulate motility, the ions themselves do not participate in this activity. Moreover, this electromotility works in a cycle-by-cycle mode up to a frequency of at least 70 kHz, which is faster than any other biological force-generator [3, 4].

Prestin belongs to a large SLC26 transporter family and constitutes a relatively novel group of protein known as the sulfate permease family, whose members are present in bacteria, fungi, plants, and animals [5]. Amino acid sequence analyses have identified prestin as the fifth member (SLC26A5) of this distinct anion transporter family. Although most members transport different anion substrates across the epithelia, prestin is unique, functioning as a voltage-dependent motor protein. Prestin is composed of a transmembrane domain, as well as of a carboxy-terminal sulfate transporter and anti-sigma factor antagonist (STAS) domain [6]. The STAS domain is critical for intracellular trafficking and protein-protein interactions [7]. OHC electromotility is accompanied by charge movement, which is characterized by a bell-shaped nonlinear capacitance (NLC) [2, 8]. NLC is regarded as the electrical signature of electromotility, which provides an intuitive readout of OHC function.

The unavailability of a prestin 3D structure signifies that most of our understanding of its mechanism is derived from experiments that use site-directed mutagenesis or domain-swapping techniques, after the amino acid residues were identified by comparing the prestin sequence to those of its paralogs and orthologs. Genome cloning of a wide range of species deduced the SLC26A5 amino acid sequences of more than 45 species [9–12]. Frog prestin is the only representative in amphibian lineage. The only study on frog prestin describes the prestin gene of *Xenopus tropicalis* (xPres) [13]. As an ancient frog species, xeno’s auditory organ is quite different from the modern frog. The present study aimed to map and sequence the SLC26A5 gene from a modern kind of frog species, *Rana catesbeiana* (rPres) via RNA sequencing (RNA-Seq) using cDNA from the frog’s inner ear. The high-throughput RNA-Seq technique, based on next-generation sequencing technology, has emerged as a useful tool for transcriptome analysis and for exploring unknown genes. RNA-Seq provides a significantly more precise measurement of transcripts than other methods and has been successfully used for gene discovery in various species [14, 15]. In order to determine if this new sequenced gene could encode a functionally active protein, we generated a stable cell line that expressed rPres. Using the whole-cell patch-clamp method, we observed that HEK293T cells expressing *Rana* prestin showed electrophysiological features similar to that of hair cells from its inner ear. Prestin functions of xeno and chick were also measured to conduct comparative study among non-mammalian species.

## Results

### rPres gene identification

In this study, we used Trinity software for the de novo assembly of reads. In total, 94,937,050 reads were obtained from the transcriptomic datas of the *Rana*’s inner ears. After discarding the low quality reads, 92,356,656 clean reads were produced with Q_30_ > 93.02%. After assembly of the transcriptome, 70.9% clean reads were successfully mapped to the *Rana* transcriptome using the Hisat software [16, 17]. Gene annotation was performed via BLAST searches of the expressed transcripts (E-value ≤10^− 5^) against the Nr (NCBI non-redundant protein sequences), Nt (NCBI nucleotide sequences), and Pfam (protein family) databases [18, 19]. The accuracy of the assembled transcriptome sequence was tested by PCR.

### Analyses of prestin orthologs

**Fig. 1:**
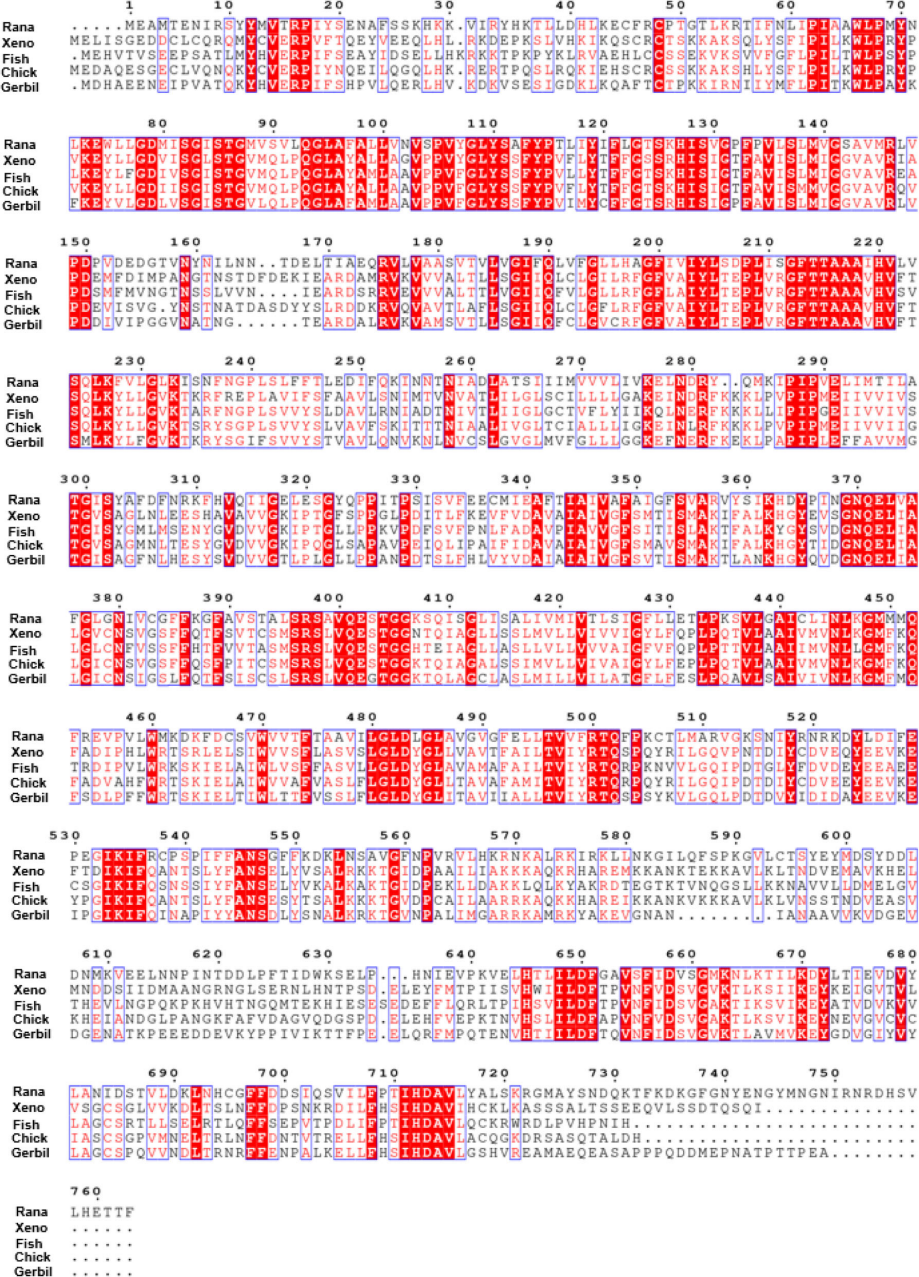
Alignment of amino acid sequences of rana (American bullfrog), xeno (tropical clawed frog), fish (zebrafish), chick and gerbil prestin. Different colors had been used to represent identity of each residue among two species. Red block: Full identity at a residue; red letter: Partial identity at a residue; Black: complete disparity at a residue. Gaps in the aligned sequences were indicated by the dashed line

We obtained the prestin coding regions of gerbil (*Meriones unguiculatus*), tropical clawed frog (*Xenopus tropicalis*), zebra fish (*Danio rerio*) and chicken (*Gallus gallus*) using BLAST analysis of the Ensembl and NCBI genomic databases. Using the CLUSTAL method, the full-length bullfrog prestin sequence mapped by RNA-Seq were aligned with the other four species [20] (Fig. 1). The results revealing an alignment with 40% identity between bullfrogs and gerbil,36% identity between bullfrogs and tropical clawed frog, 38% between bullfrogs and zebrafish and 37% between bullfrogs and chick.

### Heterologously-expressed frog prestin is localized in the cell membrane

**Fig. 2:**
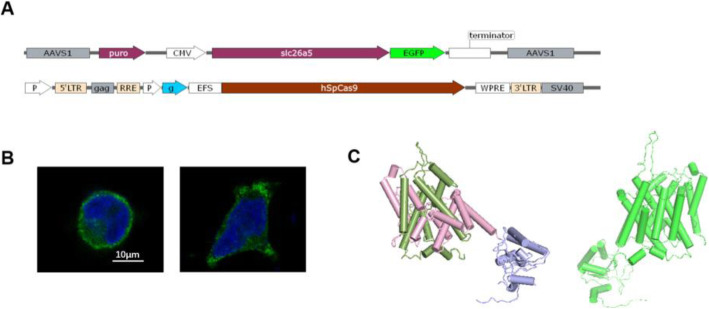
Frog prestin localized in the cell membrane. **A** Donor vector (upper one) designed for expression of ranaprestin-EGFP fusion protein and CRISPR/Cas9-mediated gene editing vector (lower one). **B** Membrane expression of rana prestin (rPres) in HEK293T cells was examined using confocal microscopy. The left one was a detached cell and the right one was an attached cell. **C** Predicted protein structure of rana prestin (left). The transmembrane domain holds two intertwined inverted repeats of seven TM segments. The N- and C-terminal halves of the TM domain are green and pink, respectively. The STAS domain is within the purple part. Predicted protein structure of gerbil prestin (right)

The function of prestin crucially relies on its integration into the cell membrane. Such localization was observed when a prestin-EGFP fusion protein was expressed in HEK293T cells. Membrane expression of the protein was examined using confocal microscopy (Fig. 2B). From the predicted 3D structure, we noticed that the frog prestin on the left side in Fig. 2C and the gerbil prestin on the right were quite similar despite their differences in the amino acid sequences. They all possessed a transmembrane domain (TM) holding two intertwined inverted repeats of seven segments and a STAS domain which was within the purple part (Fig. 2C).

### rPres confers NLC to HEK293T cells

**Fig. 3:**
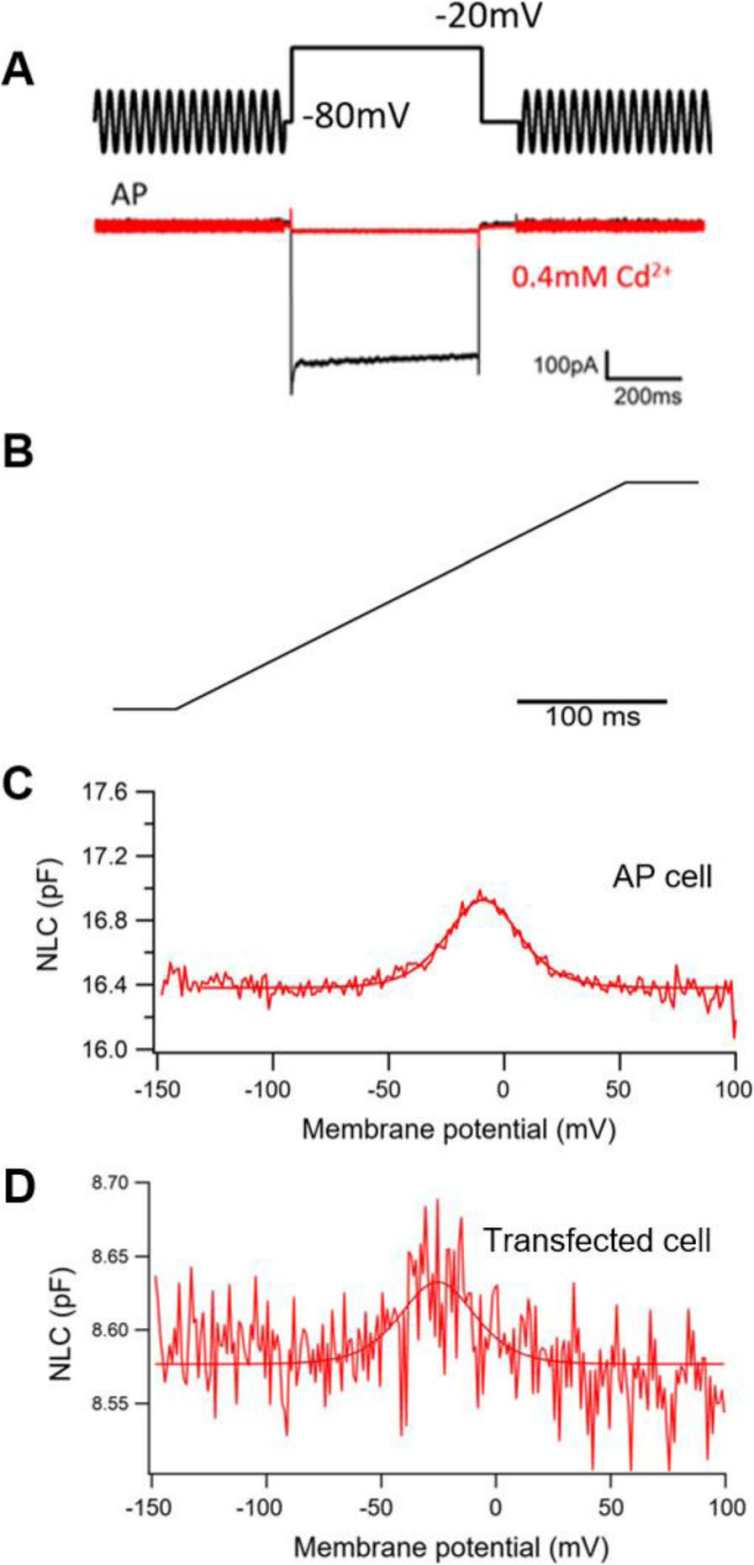
The nonlinear capacitance measurements in the hair cells from bullfrog’s amphibian papilla (AP) and in the exogenous expressed cells. **A** The voltage-clamped hair cell was depolarized from a holding potential of -80 mV to -20 mV. During the depolarizing stimulus, a Ca^2+^ current was recorded. After the Ca^2+^ current was blocked by the Cd^2+^ at a concentration of 0.4 mM. **B** Voltage steps (300 ms in duration) varied from − 150 to 100 mV in 10 mV steps were used for capacitance recordings. **C** Non-linear capacitance obtained from the hair cells of rana’s AP and **D** from the rana prestin transfected cells

The voltage-clamped frog hair cell was depolarized from a holding potential of − 80 mV to − 20 mV . During the depolarizing stimulus, the Ca^2+^ current was recorded (Fig. 3A). Voltage steps (300 ms in duration) varying from − 150 to 100 mV, in 10 mV steps, were used for NLC recordings after the Ca^2+^ current was blocked by Cd^2+^ at a concentration of 0.4 mM (Fig. 3B). NLC from HCs and transfected cells was measured using a voltage stimulus consisting of a sine wave superimposed onto a voltage ramp. Using the first derivative of the Boltzmann function, four parameters (Q_max_, C_lin_, V_1/2_, and z) from nonlinear curve-fitting of the NLC were calculated. HEK293T cells varied in size, which is correlated to the C_lin_ value. We therefore normalized Q_max_ to C_lin_ in order to compare the magnitude of the charge movement measured from cells of different sizes.

**Fig. 4:**
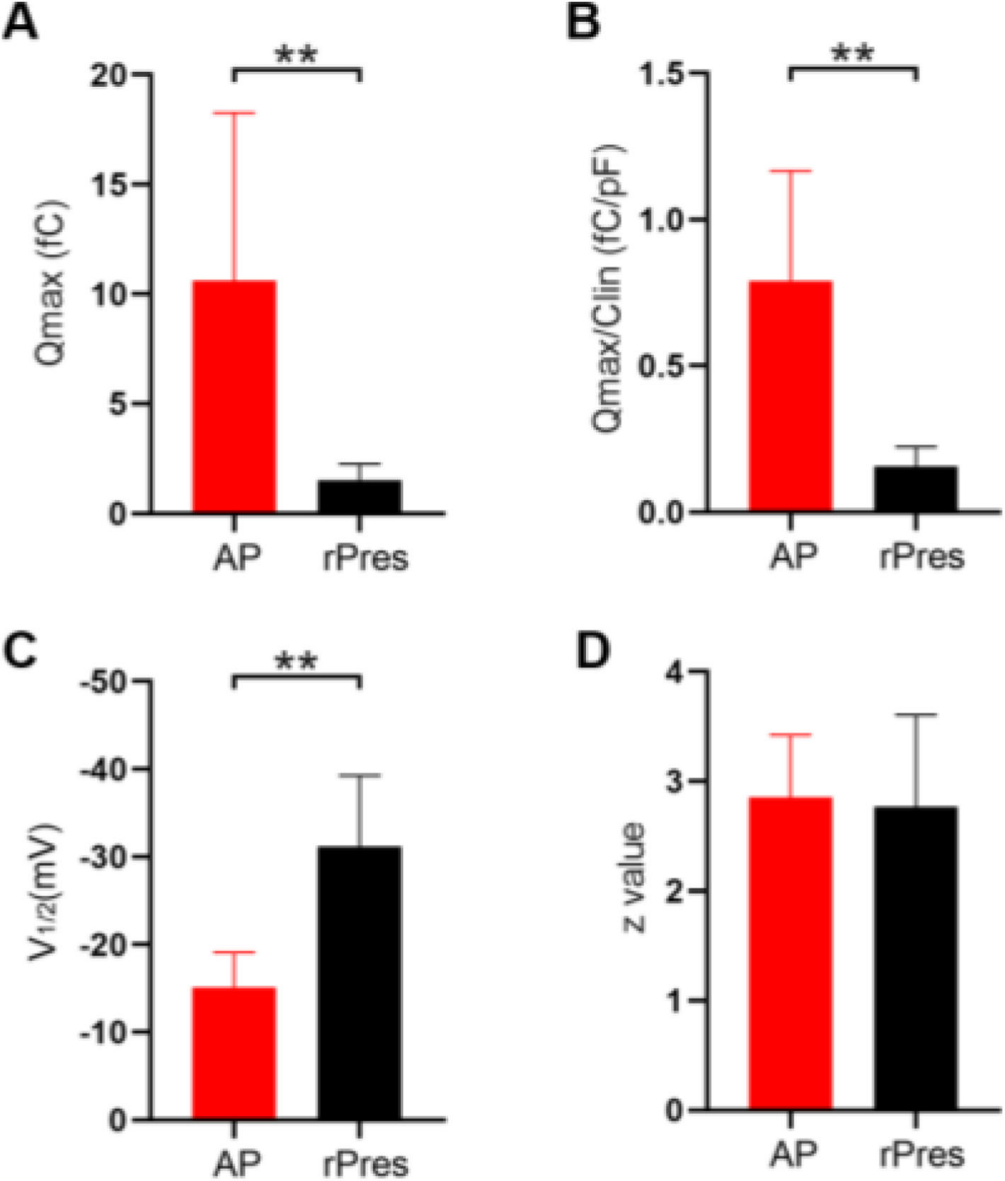
NLC datas of rana’s amphibian papilla (AP) hair cells and rana prestin transfected cells (rPres). **A**, **B**, **C**, **D** Showed four parameters derived from curve fittings with Boltzmann’s function for AP hair cells (*n* = 8) and rPres (*n* = 8). Datas were expressed as mean ± s.d. *P < 0.05, **P < 0.01

**Fig. 5:**
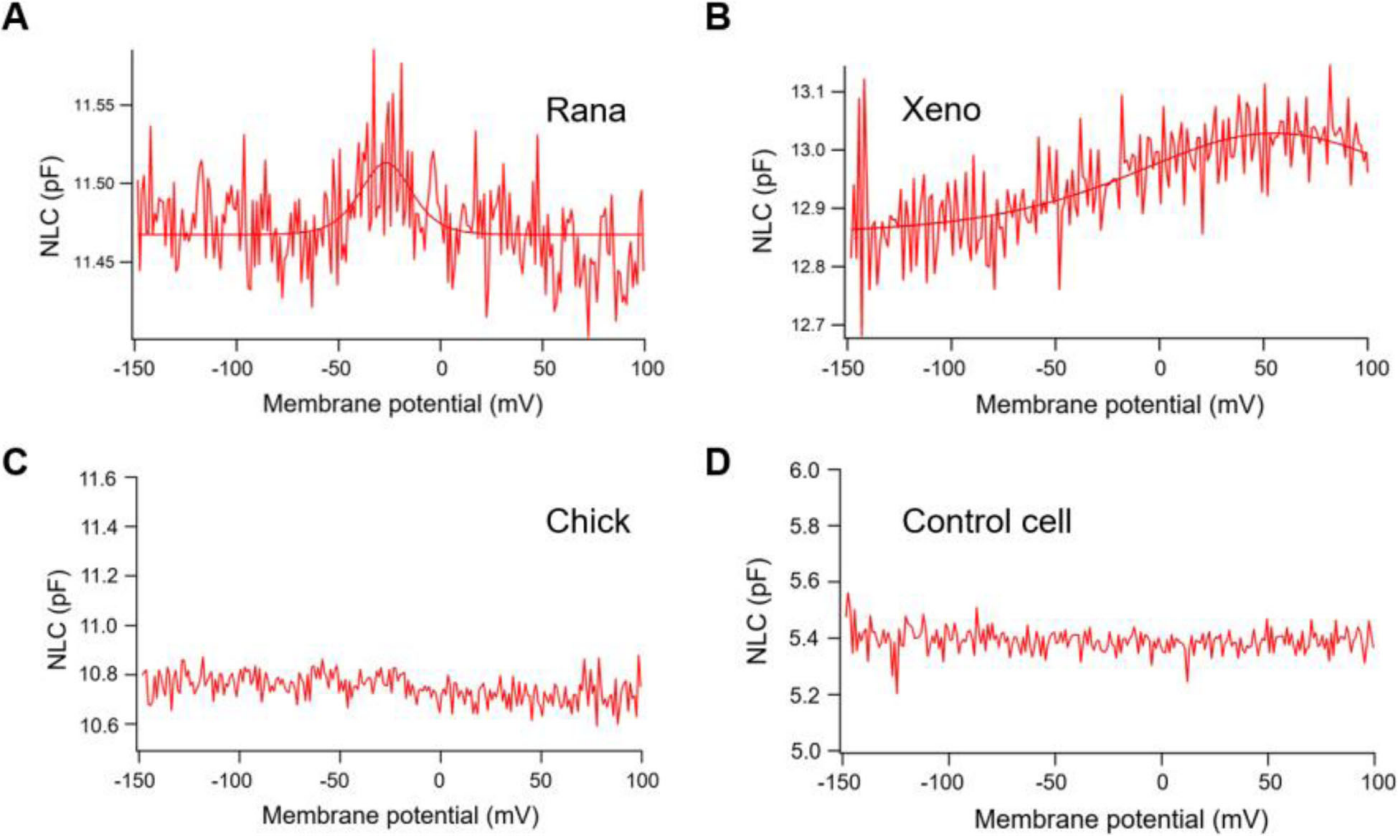
NLC measurements from rana, xenopus and chick. **A** Non-linear capacitance obtained from the HEK293T cells expressing rana prestin. **B** Non-linear capacitance obtained from the HEK293T cells expressing xenopus prestin. **C** No NLC obtained from the HEK293T cells expressing chick prestin. **D** This one showed the lack of detectable NLC in a negative control cell

NLC measurements were analyzed from eight frog HCs (Fig. 3C) and eight rPres-transfected HEK293T cells (Fig. 3D). The mean and SD values of rPres were: Q_max_ = 1.5 ± 0.7 (fC), Q_max_/C_lin_ = 0.15 ± 0.07 (fC/pF), V_1/2_ = − 31.1 ± 8.1 (mV), z = 2.8 ± 0.8. The mean and SD values of frog HCs were: Q_max_ = 11.1 ± 7.7 (fC), Q_max_/C_lin_ = 0.79 ± 0.4 (fC/pF), V_1/2_ = − 15 ± 4 (mV), z = 2.9 ± 0.6. The charge density of rPres NLC recorded from HEK293T cells was less than that from frog HCs (Fig. 4A, B; *P* < 0.01, Student’s *t*-test). Another notable functional parameter was V_1/2_ (Fig. 4C), which was significantly more hyperpolarized in rPres-transfected cells than in frog hair cells. Moreover, no significant difference was observed in the z value between the two cell types (Fig. 4D). We detected no NLC in cells transfected with the EGFP-vector only (Fig. 5D).

**Fig. 6:**
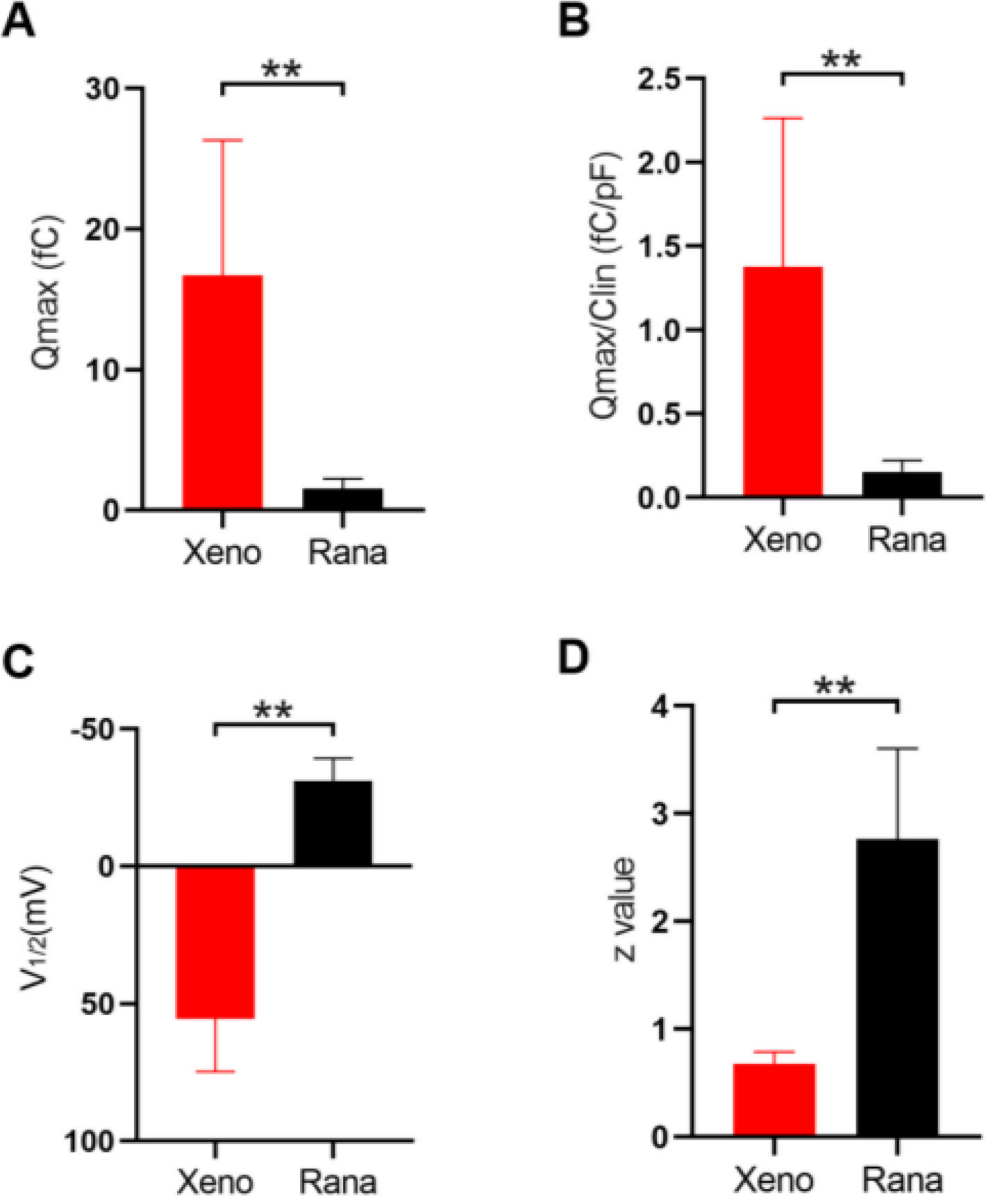
Comparing the NLC datas of xenopus prestin with rana prestin. **A**, **B**, **C**, **D**) Showed four parameters derived from curve fittings with Boltzmann’s function for the cells (*n* = 12). Datas were expressed as mean ± s.d. *P < 0.05, **P < 0.01

NLC was also measured from two other species, Xenopus (xPres) and chicken (cPres) (Fig. 5B, C). Interestingly, both amphibian species generated NLC curves. The rPres charge density was considerably less than that of xPres (Fig. 6A, B; *P* < 0.01, Student’s *t*-test), while V_1/2_ and z values varied markedly between the two (Fig. 6C, D; *P* < 0.01, Student’s *t*-test). NLC was undetected in cells transfected with cPres (Fig. 5C). All the data are shown in Table 1.

**Table 1:**
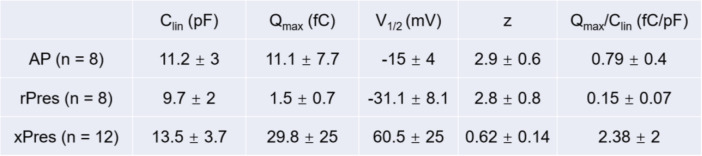
All the measurements performed in the present study are expressed as mean ± sd

## Discussion

Brownell discovered that mammalian OHCs were able to alter their length when electrically stimulated [21]. Following the discovery of “electromotility”, the study of its mechanism and role in the vertebrate auditory system became one of the most exciting areas in hearing research. Experiments which detected cellular motility even after the degradation of the cell’s content via internal tryptic digestion suggested that a molecular motor in the plasma membrane drives the mechanism of force generation [22]. The surface area of the plasma membrane was covered to nearly 70% by prestin. OHCs demonstrate piezoelectric properties with an efficiency of conversion from mechanical force to electrical charge is approximately four times greater than that of the best man-made piezoelectric material [23].

Prestin typically shares the protein structure of the SLC26A family: a conserved central region of hydrophobic amino acids with N- and C-terminal residues on the cytoplasmic side of the plasma membrane. The sulfate transporter (SulTP) sequence is located in the hydrophobic core, while a STAS domain with clusters of charged residues is present in the C-terminal region. Amino acids in the SulTP domain are almost identical among mammalian species, such as humans, mice, rats, and gerbils [24]. Although prestin was identified 20 years ago, its experimental 3D structure is still unavailable. Prestin may contain specific domains that serve as the ‘voltage sensor’ (to detect voltage change) and the ‘actuator’ (to generate length change and force). Their fundamental characteristics and mechanisms, however, remain unexplored. Approximately 200 amino acid residues have since been mutated, to determine the mechanism of action in the voltage sensor and identify sequences critical for prestin function [25]. Another approach to probe the region responsible for motor capability involves locating residues that are conserved in mammalian prestin, but variable in non-mammalian prestin orthologs. To further understand the molecular and cellular mechanisms underlying this mysterious motor protein, we attempted the mapping, sequencing, and cloning of a non-mammalian prestin ortholog using RNA-Seq.

The bullfrog SLC26A5 cDNA is 2292 bp long and encodes a predicted polypeptide of 763 amino acid residues. After isolating the prestin gene from the inner ear cDNA of the American bullfrog, we generated a stable cell line transfected with this new coding gene. Confocal images localized the heterologously-expressed frog prestin in the plasma membrane (Fig. 1B). NLC was measured both in HCs from the frog’s inner ear (Fig. 3A–C), and in HEK293T cells expressing frog prestin, to analyze its functional property (Fig. 3D). For each cell type, four parameters (Q_max_, C_lin_, V_1/2_, and z) of NLC were calculated. The charge density in frog HCs was higher than that recorded in HEK293T cells (Fig. 4A, B). Based on the fundamental assumption that a direct relationship exists between the molecular density of the protein in the cell membrane and the amount of charge recorded by the electrode [26], it is reasonable to conclude from our results that the density of endogenously-expressed prestin is higher than when expressed in the cell line. The z values obtained here should be noted (Fig. 4D). The absence of a significant difference in the z value between the two cell types suggests that the same charge is moving through the transmembrane electrical field within the protein. Alterations in intracellular ion concentration can shift the V_1/2_ direction within a range of − 180 and greater than 100 mV [27]. We observed a shift in V_1/2_ to a more positive direction from the frog AP cells, due to variations in intracellular conditions between them (Fig. 4C). Our experiment demonstrated that the new coding gene could encode a functionally active protein conferring NLC to both frog HCs and the mammalian cell line. As previous studies have shown that non-mammalian prestin does not demonstrate motor capability, and that the motor function of prestin is a newly derived molecular property exclusive to mammals [9, 13, 28], we did not attempt to examine the motor capability of frog prestin in the present study.

Analysis of the gerbil and bullfrog prestin amino acid sequences revealed approximately 40% identity among two species (Fig. 1).. Zebrafish prestin carries more than 50% sequence identity compared to that of mammals and only 38% to *Rana* (Fig. 1). As the voltage sensing range of zebrafish prestin is not within the range of –150 mV to 100 mV, uncertainty remains as to whether a two-state Boltzmann function is appropriate for its description [28]. There were 37% identity in amino acid sequences between bullfrogs and chick. Studies on avian species revealed that there were two types of HCs in the chick inner ear, neither of which possessed voltage-dependent non-linear capacitance [9]. However, contrasting results from immunolabeling studies by Maryline Beurg confirmed the presence of chick prestin in the hair cell lateral membrane, and demonstrated that HCs of the chick auditory papilla possessed NLC [29]. NLC from cPres-expressing cell lines was not detected in our study (Fig. 5C). Alignment of bullfrog, zebrafish and chick prestin showed low similarity in their amino acid sequences. The electrophysiological characteristics of their prestin were thus quite different. If the functional evolution of prestin is characterized by a gradual gain of NLC as demonstrated in a previous study, frog prestin would therefore be evolutionarily more advanced than avian and teleost prestin [11, 13].

Previous studies focusing on amphibian prestin are quite rare, let alone the knowledge of its function. Here we measured two frog species their NLC. Unlike *Rana*, xenopus is a representative of the ancient amphibian species. It’s auditory organ is different from that of *Rana*. However, it also has prestin (xPres) and its comparison with *Rana* prestin showed an identity of 36% in amino acid sequences. The measurements from xPres-expressing cell lines revealed that xPres possessed NLC (Fig. 6). The Q_max_/C_lin_ of xenopus prestin was higher than *Rana*. And the V_1/2_ was a positive value compared to *Rana*’s negative V_1/2_ value. Many studies have shown that the shift of V_1/2_ toward negative potentials may suggest an improvement in anion-binding capability [13]. Xeno prestin functions best when the transmembrane potential depolarizes to a positive point which is hard to reach. It leads to the hypothesis that the frog prestin is evolutionarily changing its function.

The presence of NLC in frog prestin might suggest a common mechanism within the protein structure for their functional significance both in mammalian and amphibian prestin. The predicted 3D structure of gerbil and frog prestin showed that they shared a similar framework (Fig. 2C). The lack of motor function in non-mammalian prestin indicates that the ‘voltage sensor’ and ‘actuator’ in the molecule may evolve independently and have different structural bases [11]. It is reasonable to assume that the voltage sensor of prestin consisted of residues present in the *Rana* prestin sequence but different in xeno and absent in zebrafish and chick prestin. Further comparative studies may reveal the molecular peculiarities underlying the mechanisms of prestin.

## Conclusion

Because data on the amphibian SLC26A5 gene is limited, we mapped and sequenced a new one from *Rana catesbeiana*’s inner earusing RNA-Seq. The *Rana*’s SLC26A5 cDNA is 2292 bp long, encoding a polypeptide of 763 amino acid residues, with 40% identity to mammals. We generated a stable cell line expressing *Rana* prestin, which possessed similar electrophysiological features as the HCs from the its auditory organ. The present study explored non-traditional species sequence information to increase our knowledge of the mechanisms involved in prestin function. Through comparative studies, we know the *Rana* prestin has been evolutionarily changing its function and becomes more advanced than avian and teleost prestin.

## Methods

### Animals

About 20 healthy adult American bullfrogs (*Rana catesbeiana*) were obtained from the same supplier (Qingpu bullfrog farm, Shanghai). Care and use of animals were conducted in accordance with the Guide for the Care and Use of Laboratory Animals (National Institutes of Health, USA) and approved by the University Committee of Laboratory Animals, Shanghai Jiao Tong University.

Ten bullfrogs weighted about 1.5 kg were sedated in an ice bath for 20 min, double-pithed, and decapitated. Inner ears of the bullfrogs were dissected and immediately immersed in RNAlater, for subsequent mRNA isolation.

### RNA isolation, library construction, and sequencing

Total RNA was isolated from the bullfrog’s inner ear using TRIzol reagent (Invitrogen, CA, USA) according to the manufacturer’s instructions. RNA quality and concentration were determined using 1.2% agarose gels and an Agilent 2100 Bioanalyzer system (Agilent Technologies, CA, USA). RNA degradation was determined using 1.2% agarose gels. RNA concentration and purity levels were measured using a NanoDrop 2000 spectrophotometer (Thermo Scientific, MA, USA). Its integrity was confirmed using an Agilent Bioanalyzer 2100 system. Sequencing libraries were generated using a NEBNext® Ultra™ RNA Library Prep Kit for Illumina® (NEB, MA, USA). mRNA was purified from 3 μg of RNA using oligo (dT) magnetic beads, and was randomly sheared into pieces of approximately 200 base pairs in the fragmentation buffer. Fragmented mRNAs were then used for first-strand cDNA synthesis using reverse transcriptase and random hexamer primers. The second-strand cDNA was synthesized using DNA polymerase I and RNase H. Following fragment ligatation to adapters, polymerase chain reaction (PCR) was used to isolate the adapter-modified fragments. The generated libraries were assessed for quality using an Agilent 2100 Bioanalyzer and a real-time PCR system, and were sequenced using an Illumina HiSeq 2500 platform (Illumina, CA, USA). Trinity (version: v2.8.4) software was used for the de novo assembly of unmapped reads, which has been efficient for the de novo reconstruction of transcriptomes from RNA-Seq data [30].

### Generation of stable cell lines that express rPres

CRISPR/Cas9-mediated gene editing was used for AAVS1 site-specific integration. The donor vector was designed to express prestin-EGFP fusion proteins (Fig. 2A, the upper one). The Cas9 vector was shown below the donor vector in Fig. 2A. The HEK293T cell line (RRID:CVCL_0063) was kindly provided by the institute of neuroscience (Chinese Academy of Sciences). Cells were cultured in Dulbecco’s modified Eagle’s medium (DMEM) (Invitrogen, Carlsbad, CA, USA) supplemented with 10% fetal bovine serum (FBS) (Invitrogen). HEK293T cells were co-transfected with the sgRNA/Cas9 and donor plasmids. Puromycin was added to the culture medium 24 h after transfection, for screening purposes. Cells transfected with the EGFP-vector were used as a negative control.

### Confocal imaging

The stable cell line was cultured for 12 h before scanned. Cells were first rinsed with phosphate-buffered saline (PBS), then fixed and permeabilized with 4% paraformaldehyde and 1% Triton X-100 for 30 min. The cells were then washed twice, each for 15 min. Confocal images were captured using a laser scanning microscope (Leica Microsystems, Germany) using a 63× oil immersion objective (Fig. 1B).

### Electrophysiology

Recordings of hair cells (HCs) from another 10 frogs were performed at 22 °C, within 2 h of dissection. Patch pipettes were of thick-walled borosilicate glass (World Precision Instruments) with a Narishige puller (model PP-830), pulled to a resistance of approximately 6 MΩ, and coated with dental wax. NLC was measured by recording dissected amphibian papillae (AP) in an extracellular solution containing (in mM): 95 NaCl, 2KCl, 1 MgCl_2_, 10 TEA-Cl, 2 CaCl_2_, 3Glucose, 1creatine, 1pyruvate and 10 HEPES at pH 7.30 (240 mOsmol L^− 1^). NaOH was used for pH adjustment. The internal solution for the HCs contains (in mM): 80Cs-gluconate, 20CsCl, 10HEPES, 2EGTA, 3 Mg-ATP, 0.5Na-GTP, 10TEA-Cl (pH 7.30; 240 mOsmol L^− 1^). CsOH was used for pH adjustment. Whole-cell voltage-clamp recordings were obtained using an EPC-10/2 (HEKA Electronics) patch-clamp amplifier and the Pulse software (HEKA). The HCs were maintained at − 80 mV, and off-line analysis was primarily using the Igor Pro 5.0 software (Wavemetrics).

HEK293T cells were detached and then bathed in an extracellular solution containing (in mM): 120 NaCl, 20 TEA-Cl, 2 CoCl_2_, 2 MgCl_2_, 10 HEPES, 5 glucose (pH 7.2). Osmolarity was adjusted to 300 mOsmol L^− 1^ with glucose. Patch pipettes were pulled to resistances of 4–6 MΩ and coated with dental wax. The internal solution was (in mM): 140 CsCl, 2 MgCl_2_, 10 EGTA and 10 HEPES. NLC measurements were performed on cultured cells with stable membrane-associated EGFP expression. After rupture, we selected cells whose membrane resistance was over 600 MΩ and showed normal Cm and Rm values. Whole-cell voltage-clamp recordings were obtained using an EPC-10/2 (HEKA Electronics) patch-clamp amplifier and the Pulse software (HEKA). The cells were maintained at − 80 mV. Off-line analysis was performed mainly using the Igor Pro 5.0 software (Wavemetrics).

The sine +DC software lock-in function of Patchmaster was used to obtain voltage-dependent currents and capacitance; a voltage protocol was designed including both ramp and sine stimulation (800 Hz with a 10-mV amplitude). Sine waves were superimposed onto ramps from − 150 mV to 100 mV for a duration of 300 ms. The NLC was fitted with the derivative of a Boltzmann function:

$$ Cm=\frac{Q_{max}\alpha }{\mathit{\exp}\left[\alpha \left({V}_m-{V}_{\frac{1}{2}}\right)\right]{\left(1+\mathit{\exp}\left[-\alpha \left({V}_m-{V}_{\frac{1}{2}}\right)\right]\right)}^2}+{C}_{lin} $$

where Q_max_ was the maximum charge transfer, V_1/2_ referred to the voltage at half-maximum charge transfer, C_lin_ represented the linear membrane capacitance, and α was the slope factor describing the voltage dependence. α = ze/kT, where k was Boltzmann’s constant, T was the absolute temperature, z was the valence of charge movement, and e was the electron charge.

### Predicting the tertiary structure of prestin

The tertiary structure of bullfrog prestin was predicted using the Phyre web server. The predicted structure was evaluated by the E value (< 0.001), which corresponding to an estimated precision over 95% [31].

### Data analysis and statistical tests

Data were analyzed in Igor Pro (WaveMetrics, USA) with home-made macros and statistical tests were performed in Prism (GraphPad, USA) with built-in functions. Depending on the nature of data set, statistical significance was assessed with unpaired Student’s t-test. Data are presented as Mean ± SD, and the level of significance was set to *p* < 0.05. In figures, ∗ means *p* < 0.05, ∗∗ means *p* < 0.01.

## Data Availability

The prestin gene IDs of gerbil (*Meriones unguiculatus*), tropical clawed frog (*Xenopus tropicalis*), zebrafish (*Danio rerio*) and chicken (*Gallus gallus*) could be accessed at GenBank under the accessions 110554811, 100497573, 322846 and 417715, respectively.

## Abbreviations

SLC26: Solute carrier family 26
NLC: Nonlinear capacitance
OHC: Outer hair cells
IHC: Inner hair cells
SulP: Sulfate permease
STAS: Sulfate transporter and anti-sigma factor antagonist
TM: Transmembrane
